# Supplementary material for: Implementation of the advanced HIV disease care package with point-of-care CD4 testing during tuberculosis case finding: A mixed-methods evaluation
Source: PLoS One. 2023 Dec 22;18(12):e0296197. doi: 10.1371/journal.pone.0296197 (PMC10745215; doi:10.1371/journal.pone.0296197)
Supplement: S1 Table — (DOCX) [file pone.0296197.s001.docx]

| S1 Table: Agreement of implementers with statements on the advanced HIV disease care package | | |  |  |  | |  |
| --- | --- | --- | --- | --- | --- | --- | --- |
|  | | | | | | |  |
|  | | | **3 months** | | **6 months** | |  |
| **Acceptability** | | | **(n=8)** | **(n=8)** |  | |  |
| 1 | Overall, I am satisfied with the AHD care package | | 1 [1-2] | 2 [1-2] |  | |  |
| 2 | I would recommend the AHD care package for primary care level | | 2 [1-2] | 1 [1-2] |  | |  |
| 3 | The AHD care package can help treat patients with advanced HIV | | 1 [1-2] | 1 [1-1] |  | |  |
| 4 | The AHD care package can improve the quality of my work | | 1 [1-1] | 1 [1-2] |  | |  |
| 5 | The AHD care package can reduce the risk of death in patients with advanced HIV | | 1 [1-2] | 1 [1-2] |  | |  |
| 6 | Some health care workers might not understand the importance of the AHD care package † | | 4 [2-4] | 2 [2-3] |  | |  |
|  |  | |  |  |  | |  |
| **Acceptability for patients** | | |  |  |  | |  |
| 7 | Patients accept the AHD care package | | 2 [2-3] | 2 [1-2] |  | |  |
| 8 | Some patients prefer not to receive the AHD package † | | 4 [2-4] | 2 [2-4] |  | |  |
| 9 | Patients understand the importance of the AHD care package | | 2 [1-2] | 1 [1-2] |  | |  |
| 10 | It is acceptable for patients to receive the tests in the community | | 1 [1-2] | 2 [2-2] |  | |  |
|  |  | |  |  |  | |  |
| **Intervention delivery** | | |  |  |  | |  |
|  | | |  |  |  | |  |
| **Enablers (and quality)** | | |  |  |  | |  |
| 11 | Health workers understand the procedures to follow for AHD implementation | | 1 [1-2] | 2 [1-2] |  | |  |
| 12 | Health workers independently perform the AHD care package | | 2 [1-3] | 1 [1-2] |  | |  |
| 13 | The procedure is sufficiently explained by the provided flow charts and job aids | | 2 [1-2] | 2 [1-2] |  | |  |
| 14 | The training was sufficient for the health workers to independently perform the package | | 2 [1-2] | 2 [1-2] |  | |  |
| 15 | The training material needs improvement for effective implementation of the AHD package † | | 3 [2-4] | 2 [2-3] |  | |  |
| 16 | The quality of implementation of the AHD care package is sufficient | | 2 [1-2] | 2 [1-2] |  | |  |
| 17 | Health care workers need more training to implement the AHD care package with acceptable quality † | | 3 [3-4] | 4 [3-4] |  | |  |
| 18 | If implemented in the community, the quality of implementation of the AHD package would be similar | | 2 [1-2] | 2 [1-3] |  | |  |
|  |  | |  |  |  | |  |
| **Challenges** | | |  |  |  | |  |
| 19 | The different steps to take in the AHD care package are complex for most health workers † | | 2 [2-3] | 2 [2-3] |  | |  |
| 20 | The time to perform all different steps is a challenge for implementation † | | 4 [3-5] | 2 [2-4] |  | |  |
| 21 | The number of different steps in the procedure is acceptable for implementation | | 2 [1-2] | 2 [2-2] |  | |  |
| 22 | All the tests for TB-Triage+, including AHD, can be administered without major problems | | 2 [1-2] | 2 [1-2] |  | |  |
| 23 | It is challenging to administer the AHD package within TB-triage + procedures † | | 2 [1-3] | 2 [1-2] |  | |  |
| 24 | The ministry of health should implement the AHD care package for primary care facilities | | 2 [1-2] | 1 [1-2] |  | |  |
| 25 | More experience in other settings is needed before nationwide implementation of the AHD care package † | | 3 [2-4] | 2 [2-3] |  | |  |
|  | | | | | | |  |
|  | | | | | | |  |
|  | **During COVID-19** | |  |  |  | |  |
| 26 | In combination with COVID-19 testing, the AHD implementation is still feasible | | 2 [1-2] | 2 [2-2] |  | |  |
| 27 | It is challenging to administer the AHD package within the context of COVID-19 † | | 2 [2-2] | 2 [2-3] |  | |  |
|  |  | |  |  |  | |  |
|  | **In a community setting** | |  |  |  | |  |
| 28 | The AHD package is feasible as part of a community TB-triage trial | | 1 [1-2] | 2 [1-2] |  | |  |
| 29 | Community test and treat campaigns are appropriate for AHD administration | | 2 [1-2] | 1 [1-2] |  | |  |
| 30 | I foresee difficulties for implementation at community test & treat campaign † | | 2 [2-4] | 2 [2-2] |  | |  |
| 31 | The ministry of health should implement of the AHD care package as part of community activities | | 2 [2-3] | 2 [1-2] |  | |  |
|  | The order of the statements has been changed compared to the original questionnaire  Participants scored their agreement with the statement as follows: I strongly agree (1), I agree (2), I’m neutral (3), I disagree (4), I strongly disagree (5). | |  |  |  | |  |
|  | † Outcomes of negatively framed statements were recoded to allow comparison: I strongly agree (5), I agree (4), I’m neutral (3), I disagree (2), I strongly disagree (1).  All statement with median scores of 3 or more were discussed in debt in group discussions. | |  |  |  | |  |
|  |  | |  |  |  | |  |
