# Supplementary material for: Implementation of the advanced HIV disease care package with point-of-care CD4 testing during tuberculosis case finding: A mixed-methods evaluation
Source: PLoS One. 2023 Dec 22;18(12):e0296197. doi: 10.1371/journal.pone.0296197 (PMC10745215; doi:10.1371/journal.pone.0296197)
Supplement: S2 Table — (DOCX) [file pone.0296197.s002.docx]

| S2 Table: Difficulty of different steps of procedure perceived by implementers of advanced HIV disease care package | | | |
| --- | --- | --- | --- |
|  | 3 months (n = 8) | 6 months (n = 8) |  |
|  | Median score (IQR) | Median score (IQR) |  |
| AlereLAM | 2 [1-2] | 2 [1-2] |  |
| VISITECT | 1 [1-2] | 1 [1-2] |  |
| Immy CrAg | 1 [1-2] | 1 [1-2] |  |
| Writing a referral letter/note for treatment | 2 [1-2] | 1 [1-1] |  |
| Ensuring the correct timing of each different step is followed | 2 [1-3] | 1 [1-2] |  |
| Reading and interpreting the different results | 2 [1-2] | 1 [1-2] |  |
| Performing regular quality controls for the tests | 1 [1-2] | 1 [1-1] |  |
| Entering correct data on the test results | 1 [1-2] | 2 [1-2] |  |
| Any part of the procedure | 1 [1-2] | 1 [1-2] |  |

Participants answered the question: How high do you estimate the risk to make a mistake in this part of the procedure? Possible answers were very low (1), low (2), neutral (3), high (4) and very high (5).

VISITECT; VISITECT CD4 Advanced Disease, Immy CrAg, Immy cryptococcal antigen lateral flow assay; IQR, interquartile range; AlereLAM, Alere tuberculosis lipoarabinomannan lateral flow assay.
