# Supplementary material for: Implementation of the advanced HIV disease care package with point-of-care CD4 testing during tuberculosis case finding: A mixed-methods evaluation
Source: PLoS One. 2023 Dec 22;18(12):e0296197. doi: 10.1371/journal.pone.0296197 (PMC10745215; doi:10.1371/journal.pone.0296197)
Supplement: S3 Table — (DOCX) [file pone.0296197.s003.docx]

# S3 Table. Summary of procedural completeness and duration of rapid tests of the advanced HIV disease care package

|  | Overall | | | | Lesotho | | | South Africa | | |
| --- | --- | --- | --- | --- | --- | --- | --- | --- | --- | --- |
| Completeness (score 1-5) | | n |  | score | n |  | score | n |  | score |
| VISITECT | | 13 |  | 5 [5-5] | 7 |  | 5 [5-5] | 6 |  | 5 [5-5] |
| AlereLAM | | 13 |  | 5 [5-5] | 7 |  | 5 [5-5] | 6 |  | 5 [4-5] |
| Immy CrAg | | 5 |  | 5 [5-5] | 3 |  | 5 [5-5] | 2 |  | 4 [3-5] |
| Duration (minutes) | | n |  | duration | n |  | duration | n |  | duration |
| HIV Test 1 | | 4 |  | 17 [16-23] | 4 |  | 17 [16-23] | 0 |  | NA |
| HIV Test 2 | | 1 |  | 21 | 1 |  | 21 | 0 |  | NA |
| VISITECT | | 12 |  | 45 [42-51] | 6 |  | 48 [44-52] | 6 |  | 43 [40-50] |
| AlereLAM | | 12 |  | 34 [31-37] | 6 |  | 34 [32-40] | 6 |  | 32 [27-34] |
| Immy CrAg^†^ | | 4 |  | 11 [10-13] | 3 |  | 10 [10-14] | 1 |  | 12 |
| VISITECT + AlereLAM | | 13 |  | 61 [48-71] | 7 |  | 61 [45-65] | 6 |  | 63 [50-74] |
| VISITECT + AlereLAM + Immy CrAg | | 5 |  | 73 [68-85] | 3 |  | 73 [52-142] | 2 |  | 77 [68-85] |
| Results registration | | 8 |  | 9 [5-11] | 5 |  | 9 [2-12] | 3 |  | 8 [7-10] |

† time to prepare plasma sample not included

A score from 0–5, signifying totally incomplete to complete was given on a five-point Likert-type scale, and the start- and end time of each procedure was collected. Rating scales were completed at two (median, IQR:2−4) months after study start, and two (median, IQR:1−2) were collected per implementer (interval 84 (median, IQR:57−89) days)

Procedures were rated incomplete in South Africa because 1) a whole blood sample was used (not plasma) for Immy CrAg 2) of uncertainty if a patient provided midstream urine for AlereLAM.

VISITECT; VISITECT CD4 Advanced Disease, Immy CrAg, Immy cryptococcal antigen lateral flow assay; NA, not applicable; AlereLAM, Alere tuberculosis lipoarabinomannan lateral flow assay
