# Supplementary material for: Implementation of the advanced HIV disease care package with point-of-care CD4 testing during tuberculosis case finding: A mixed-methods evaluation
Source: PLoS One. 2023 Dec 22;18(12):e0296197. doi: 10.1371/journal.pone.0296197 (PMC10745215; doi:10.1371/journal.pone.0296197)
Supplement: S4 Table — (DOCX) [file pone.0296197.s004.docx]

# S4 Table: Logistic regression of predictors for being alive at 12 weeks

| **Variable** | |  | Univariable analysis | | | |  | Multivariable model 1 | | | |  | Multivariable model 2 | | | |
| --- | --- | --- | --- | --- | --- | --- | --- | --- | --- | --- | --- | --- | --- | --- | --- | --- |
|  |  | | OR | 95% CI | | p-value |  | aOR | 95% CI | | p-value |  | aOR | 95% CI | | p-value |
| AHD status | No AHD | | 1 |  |  |  |  | 1 |  |  |  |  | NA |  |  |  |
|  | AHD † | | 0.48 | 0.29 | 0.81 | 0.005 |  | 0.71 | 0.40 | 1.27 | 0.245 |  | NA |  |  |  |
|  | Unknown | | 2.54 | 0.59 | 10.99 | 0.212 |  | 1.84 | 0.41 | 8.16 | 0.424 |  | NA |  |  |  |
| Composite TB result | Negative | | 1 |  |  |  |  | NA |  |  |  |  | 1 |  |  |  |
|  | Positive ‡ | | 0.90 | 0.50 | 1.62 | 0.726 |  | NA |  |  |  |  | 1.10 | 0.59 | 2.07 | 0.762 |
|  | Unknown | | 4.49 | 1.07 | 18.87 | 0.040 |  | NA |  |  |  |  | 2.93 | 0.68 | 12.63 | 0.149 |
| Age |  | | 1.00 | 0.98 | 1.02 | 0.705 |  | 1.01 | 0.99 | 1.03 | 0.463 |  | 1.01 | 0.99 | 1.03 | 0.397 |
| Sex | Female | | 1 |  |  |  |  | 1 |  |  |  |  | 1 |  |  |  |
|  | Male | | 0.47 | 0.28 | 0.79 | 0.005 |  | 0.60 | 0.33 | 1.08 | 0.089 |  | 0.58 | 0.32 | 1.05 | 0.072 |
| Country | South Africa | | 1 |  |  |  |  | 1 |  |  |  |  | 1 |  |  |  |
|  | Lesotho | | 0.37 | 0.22 | 0.63 | 0.000 |  | 0.45 | 0.25 | 0.80 | 0.007 |  | 0.44 | 0.25 | 0.77 | 0.004 |
| HIV status | Known HIV positive on ART | | 1 |  |  |  |  | 1 |  |  |  |  | 1 |  |  |  |
|  | Newly diagnosed | | 0.98 | 0.38 | 2.58 | 0.975 |  | 1.78 | 0.57 | 4.36 | 0.379 |  | 1.43 | 0.51 | 3.98 | 0.492 |
|  | Known HIV positive not on ART | | 0.40 | 0.17 | 0.91 | 0.029 |  | 0.82 | 0.33 | 2.00 | 0.656 |  | 0.72 | 0.29 | 1.76 | 0.470 |
| BMI |  | | 1.07 | 1.02 | 1.13 | 0.006 |  | 1.21 | 0.96 | 1.09 | 0.505 |  | 1.03 | 0.97 | 1.09 | 0.397 |
| TB symptom | Cough | | 0.43 | 0.13 | 1.42 | 0.165 |  | 0.49 | 0.14 | 1.65 | 0.250 |  | 0.50 | 0.15 | 1.68 | 0.260 |
|  | Fever | | 0.73 | 0.42 | 1.28 | 0.269 |  | NA |  |  |  |  | NA |  |  |  |
|  | Weight loss | | 0.58 | 0.34 | 0.97 | 0.039 |  | 0.76 | 0.42 | 1.37 | 0.361 |  | 0.73 | 0.41 | 1.30 | 0.279 |
|  | Night sweats | | 0.87 | 0.53 | 1.42 | 0.575 |  | NA |  |  |  |  | NA |  |  |  |

†CD4 ≤200cells/µl or AlereLAM positive or MTB detected on Xpert MTB/RIF or Xpert MTB/RIF Ultra or MGIT culture

‡ MTB detected on Xpert MTB/RIF. Xpert MTB/RIF Ultra or MGIT culture or AlereLAM

AHD, advanced HIV disease; aOR, adjusted odds ratio; ART, antiretroviral treatment; BMI, body-mass index, CI, confidence interval; OR, odds ratio; TB, tuberculosis
